# Advancing precision diagnostics: minimally invasive approaches for understanding the role of brain-limited somatic mutations in pediatric drug-resistant epilepsy

**DOI:** 10.3389/fsurg.2025.1568939

**Published:** 2025-05-23

**Authors:** Olubunmi A. Fariyike, Nishanth Narayan, Hilary Y. Liu, Danielle R. Sanchez, H. Westley Phillips

**Affiliations:** ^1^ Stanford University School of Medicine, Stanford University, Stanford, CA, United States; ^2^ Department of Neurosurgery, Stanford University, Stanford, CA, United States; ^3^ University of Pittsburgh School of Medicine, University of Pittsburgh, Pittsburgh, PA, United States; ^4^ Department of Biological Sciences, University of California, Davis, Davis, CA, United States

**Keywords:** cerebrospinal fluid, drug-resistant epilepsy, genetics, implanted electrodes, minimally invasive surgical procedures, molecular pathology, neurosurgery, pediatrics

## Abstract

For the one-third of epilepsy patients whose disease is refractory to medical therapies, the social, economic, and developmental consequences are often devastating and lifelong. This has sparked great interest in the elucidation of the genetic drivers of epilepsy for the discovery of precision therapies. Over the past 30 years, tissue derived from standard-of-care open resections has provided genetic material for a wealth of research on the genetic mechanisms of epileptic disease. One of the most important findings of this research is the presence of pathogenic brain-limited somatic mutations; however, many patients who would benefit from genetic analysis are not surgical candidates. Further, as minimally invasive techniques such as laser ablation and neuromodulation become increasingly indicated, access to surgically resected brain tissue may become more limited, posing challenges for the research and diagnostic advancements that have traditionally relied on such samples. Fortunately, two minimally invasive methods for obtaining brain-derived genetic material have been developed in recent years. Both cell-free DNA isolated from cerebrospinal fluid and DNA extracted from microbulk tissue adherent to stereo-EEG (sEEG) electrodes have demonstrated sufficient quantity and quality for identification of brain-limited somatic variants. Both techniques have important advantages over surgically obtained bulk-brain tissue and hold promise as new leading avenues of genetic epilepsy research. This article provides a general overview of brain-limited somatic variants in pediatric drug-resistant epilepsy, with a specific focus on the evidence for the use of electrode- and cerebrospinal fluid-derived DNA. We also detail the specific advantages and disadvantages of these minimally invasive techniques as compared to the use of traditional, resection-derived bulk tissue.

## 1 Introduction

Approximately 50 million people globally are estimated to live with epilepsy, a heterogenous set of disorders characterized by recurrent and often difficult-to-predict seizures (1). Notably, among the pediatric population, epilepsy is the most common chronic neurological disease, affecting up to one percent of all children (2, 3). Of this one percent, approximately one-third will have drug-resistant epilepsy (DRE), defined as the persistence of seizures after two properly trialed and tolerated anti-seizure medications (ASMs) (4). Once diagnosed with DRE, the chances of seizure freedom using ASMs alone diminishes precipitously (5–7). Children with epilepsy are at higher risk for poor academic performance, difficulty with socialization throughout childhood and into adulthood, and overall mortality, especially for those who do not eventually achieve seizure freedom (8–16). This poor prognosis has spurred decades of research into the genetic mechanisms underlying epilepsy, with the hopes of developing more effective treatments, more accurate prognoses, and hopefully, an eventual cure.

Our understanding of epilepsy as a genetic rather than clinical disease began as early as the 1940s with twin studies that suggested a heritable predisposition towards epilepsy (17). And yet, possible genetic mechanisms driving said heritability were not reported until the landmark 1995 discovery of a monoallelic *CHRNA4* missense mutation that conferred autosomal dominant nocturnal frontal lobe epilepsy in one family (18). This finding was the catalyst for the discovery of several other monogenic epilepsy syndromes in the following decades (19). Germline variants such as these are present in all bodily tissues and are therefore readily detectable in commonly available clinical samples, such as saliva or blood. Nevertheless, germline variant identification using such samples failed to provide genetic diagnoses for many DRE patients (19, 20). Thus, borrowing from the field of oncology, epilepsy researchers began to take interest in *de novo* somatic mutations as a possible additional genetic mechanism for explaining epileptic pathophysiology (21).

**Figure 1:**
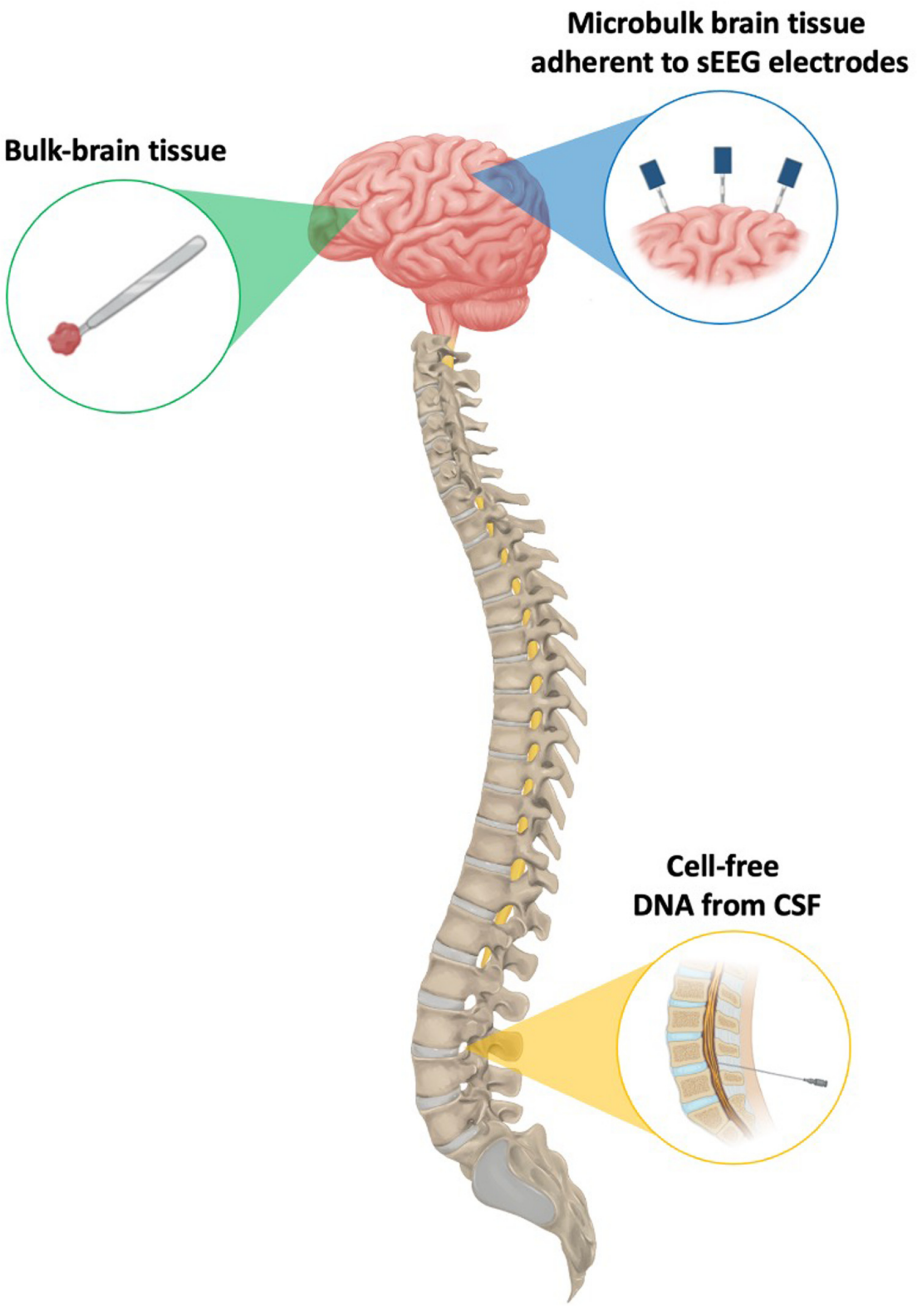
A visual representation of the different sources of genetic material that have been leveraged for the successful detection of brain-limited somatic variants.

**Figure 2:**
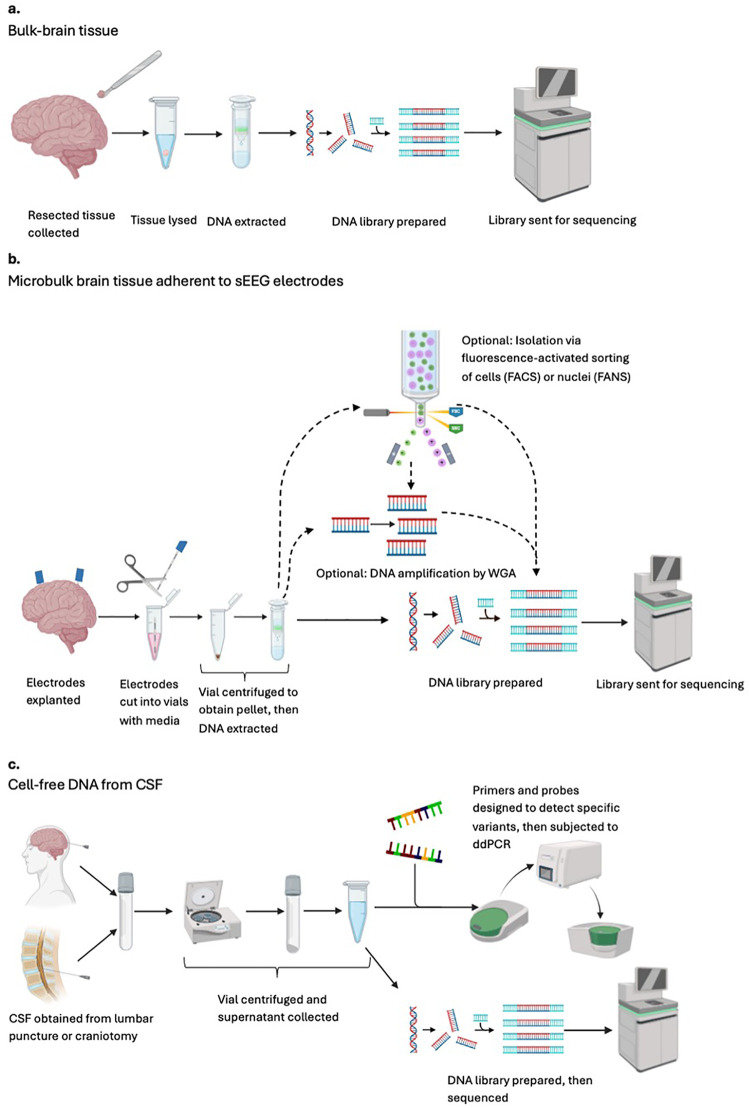
An overview schematic of the sample processing and analysis pipelines available for identification of genomic variants in pediatric drug-resistant epilepsy. Each row indicates a different general workflow when the starting material is **(a)** bulk-brain tissue **(b)** microbulk brain tissue adherent to sEEG electrodes, and **(c)** cell-free DNA from CSF. CSF, cerebrospinal fluid; FACS, fluorescence-activated cell sorting; FANS, fluorescence-activated nuclei sorting; sEEG, stereo-electroencephalography (stereo-EEG); WGA; whole-genome amplification.

Unlike germline variants, somatic variants arise after zygote formation. This leads to “somatic mosaicism,” defined as the presence of a mutation at differing allele fractions both within and between tissues that may cause disease in the affected individual without being passed onto offspring (19). Therefore, the ability to detect a somatic variant depends largely on when in development and in what parent cell the original mutation occurred. For example, a mutation that occurs before gastrulation may be present across several tissues of different embryonic origins, thus easily detected in blood or saliva. Conversely, a mutation originating later in development in a neural tube cell destined to form part of the brain is limited to the brain itself, with more brain tissue harboring the mutation the earlier in development the mutation occurred. The focus of this review is this latter type of brain-limited somatic mutation. Given their brain-restricted nature, direct sampling of brain tissue or another source of brain-derived DNA is necessary for variant identification (Figure 1). Traditionally, this has required open epilepsy surgery, with collected tissue subjected to cell lysis and DNA extraction (Figure 2a). Extracted DNA can then be used for different forms of next-generation sequencing (NGS), including targeted panel, whole-exome, and whole-genome sequencing. The general considerations for each sequencing strategy are well-described (22, 23). Analysis of any of the aforementioned sequencing workflows yields candidate variants, whose potential for pathogenicity can then be determined using established pipelines (24–29).

Open surgical resection is a mainstay of DRE management, especially in cases for which suspected causative lesions are identified on imaging as well as in temporal lobe epilepsy, where postoperative seizure freedom rates as high as 83% have been reported (30–34). This resected tissue has led to several advances in the classification of different epilepsy syndromes by shared genetic mechanisms, which are excellently summarized elsewhere (19, 35, 36). Beyond a basic science understanding, however, this research has also created the foundation for precision medicine in epilepsy. For example, the pivotal Examining Everolimus in a Study of Tuberous Sclerosis Complex (EXIST-3) trial demonstrated the efficacy of everolimus, a mammalian target of rapamycin (*mTOR*) inhibitor, in reducing seizure burden among patients with tuberous sclerosis complex (TSC). This discovery led to the drug's U.S. Food and Drug Administration (FDA) approval for use in TSC patients in 2018 (37, 38). On the other hand, commonly used ASMs such as carbamazepine and phenytoin, whose mechanisms of action involve inhibition of sodium channels, have been shown to be contraindicated in *SCN1A*-related channelopathies, such as Dravet syndrome, due to increased seizure risk (39, 40).

To date, our understanding of brain-limited somatic variants has largely depended on resected tissue. However, as all fields of surgery–including neurosurgery–begin to see increasing indications for minimally invasive surgical approaches given their reduced costs and lower risk profile, maintaining access to epileptic brain tissue and/or its component genetic material will be paramount to advancing molecular diagnosis and precision therapies (41, 42). Therefore, this review will specifically focus on alternative methods for elucidating brain-limited somatic mutations driving epilepsy without the use of resection-derived tissue. We will briefly summarize the limitations of open resection as the exclusive method for variant discovery before in-depth discussion of two emerging minimally invasive approaches: isolating cell-free DNA (cfDNA) from cerebrospinal fluid (CSF) and collecting microbulk tissue from stereo-EEG (sEEG) electrodes (Figure 1). Finally, we will present the challenges and opportunities associated with each of these new methods.

## 2 Limitations of resected tissue as the only source of genetic material

Even when considering the research advances made using bulk-brain tissue from open resection, there are several limitations to its use as the sole means of elucidating genetic mechanisms in epilepsy. Most critically, waiting until resection for genetic diagnosis limits our ability to provide rapid and personalized medical therapy until after an invasive procedure has been performed. During this time, patients live with uncontrolled seizures and their known detrimental effects on growth and development as well as current and future social and economic life (9–11, 43–47). Additionally, under this surgical diagnostic paradigm, patients who are not surgical candidates due to comorbidities or lack of a definitive surgical target after presurgical evaluation cannot receive an exhaustive genetic workup beyond blood-based diagnostic tests, which are incapable of discovering brain-limited somatic mutations. Lastly, our reliance on surgical samples limits genetic analysis to regions that are surgically accessible and indicated for resection. Therefore, the role of mutations in regions outside of the resection zone remains understudied and poorly understood.

## 3 CSF and sEEG electrodes as promising additional sources

### 3.1 Microbulk tissue from sEEG electrodes

When surgical management is being considered for a patient with DRE, there are a variety of non-invasive imaging and electrophysiological techniques for localizing suspected epileptic regions. However, when these modalities are discordant or the putative epileptogenic zone abuts brain regions directly implicated in brain function, more invasive methods are often indicated for definitive resolution of a surgical target. Of these more invasive techniques, sEEG is a minimally invasive option that offers the benefits of operative versatility and reduced procedural complications, with clinical outcomes comparable to existing subdural grids and strips (48–51). When electrodes are explanted days to weeks after implantation, some cells remain adherent to the portions directly in contact with the brain, providing a source of microbulk brain tissue from which DNA can be extracted for NGS and subsequent identification of brain-limited somatic variants (Figure 1) (52–59).

In general, the workflow begins with electrode explantation (Figure 2b). Collected electrodes can be frozen indefinitely or used immediately. Some groups will additionally subject samples to whole-genome amplification (WGA) to increase DNA yield before sequencing. When possible, electrode samples are sequenced alongside matched blood to facilitate the differentiation of germline and multi-tissue somatic variants–both of which are expected to also appear in blood–from brain-limited somatic mutations. Sequencing data from a set of normal controls can also be used to identify and exclude common population-level variants. This technique was first described in 2019 when Montier and colleagues identified a novel, *de novo,* heterozygous, frameshift *MEN1* mutation in an adult male patient suffering from DRE secondary to periventricular nodular heterotopia (PVNH). Previous blood-based genetic analyses for detection of germline variants had been unrevealing. In this pioneering study, all electrode-derived cell pellets were pooled before WGA, resulting in an overall variant allele frequency (VAF) of 16.7% (52).

Three years later, Ye and colleagues were the first to demonstrate a somatic gradient in an adult woman with nonlesional, multifocal epilepsy. Electrodes were divided into three pools by brain region before WGA, and a loss-of-function variant in the *KCNT1* gene was identified. The highest VAF corresponded to the most epileptogenic region by both scalp and stereo-EEG (53). In 2024, Klein and colleagues also published a case report, revealing a mosaic missense *mTOR* variant in a pediatric patient with focal cortical dysplasia (FCD). Nuclei isolated from five electrodes were sorted via fluorescence-activated nuclei sorting (FANS) before WGA and NGS. The aforementioned variant was found exclusively in neuronal nuclei of the affected region at a VAF of only 0.78% and not in unaffected brain regions, astrocytes, or saliva (54). Gatesman and colleagues similarly reported the discovery of a mosaic *FGFR1* mutation from a single electrode inserted into known tumorous tissue in a male pediatric patient with a low-grade epilepsy-associated tumor (LEAT). This variant was not identified in previous blood-based genetic analyses and was verified by Sanger sequencing (55).

On the other hand, Checri and colleagues analyzed individual electrodes for multiple patients. Uniquely, they used unamplified, electrode-derived DNA from three pediatric FCD patients with known variants identified in prior resection-derived tissue analysis. For two of the three patients, these known, tissue-derived variants were also identified from electrodes, although at lower VAFs. Of the 33 analyzed electrodes, variants were confirmed in four. Three of these electrodes were located in the epileptogenic zone of one patient and one in the propagation zone of the other (56). Shortly after, Phillips and colleagues analyzed individual electrodes from a single patient, establishing a gradient for a somatic point *PIK3CA* mutation in a female with a known malformation of cortical development (MCD). VAFs for five electrodes and for tissue biopsies collected near each electrode entry site were inversely correlated with distance from the seizure onset zone. Notably, compared to tissue samples, the variant was found at lower VAFs in the electrodes, but with similar gradient topography. In short, for both sample types, the mutational burden was highest in areas implicated in seizure onset and lowest in areas that were electrophysiologically normal. Conversely, this mutation was absent from all unamplified electrode-derived DNA samples, suggesting the utility of WGA for detection of some rare somatic variants when using sEEG-derived DNA (57). In a similar fashion, Mascarenhas and colleagues analyzed individual electrodes from 17 pediatric patients. FANS was used to isolate neuronal nuclei for WGA, and electrode samples were subjected to both pre-sequencing short-tandem repeat analysis and post-sequencing allelic imbalance analysis as forms of quality control. Samples from seven patients were ultimately analyzed, revealing pathogenic somatic variants in the *mTOR*, *CSDE1*, *KLLN*, and *NLE1* genes across four patients (58). Most recently, building upon their previous case report (57), D'Gama and colleagues analyzed ten additional pediatric DRE patients, the largest cohort to date subjected to sEEG-based genetic analysis. WGS was performed on individual electrodes as well as on resection-derived tissue and whole blood, when available. Concurrent immunohistochemical studies confirmed the presence of neuronal tissue. Ultimately, four pathogenic or likely pathogenic variants—one each in the *CNTNAP2*, *CIC*, *PTEN*, and *KDM6A* genes—were found in three patients who had undergone laser ablation, and therefore, did not have resected tissue available for analysis. They were also able to detect previously identified germline *TSC2* and *DEPDC5* variants in two additional patients (59).

Despite various studies demonstrating that a portion of electrode-derived cells possess neural markers, uncertainty remains concerning which exact cell types and brain regions are represented in an electrode sample (53, 54, 58, 59). The possible dilution of neural cells with other inflammatory or hematopoietic cells has been partially addressed by using fluorescence-activated isolation for cell- or nuclei-specific enrichment (54, 58). Nevertheless, electrodes are explanted by pulling along their initial implant trajectory. Therefore, whether subsegments within a given explanted electrode recapitulate the unique genetic profiles of the specific brain regions in which they rested or instead reflect an average profile of all traversed brain regions is an area of active investigation. Overall, however, electrode-derived DNA shows promise in facilitating a more complete evaluation of electrophysiological and genetic profiles as well as their interplay throughout more regions of the brain than previously possible (53).

### 3.2 Cell-free DNA from CSF

CSF contains another source of trace genetic material that can be used to investigate brain-limited somatic variants. Cell-free DNA (cfDNA) refers to short DNA sequences approximately 150 base pairs in length that are released into the circulation upon cell death and can be isolated from urine, peripheral blood, saliva, and even CSF (Figure 1) (60). cfDNA was first utilized in a 2010 breast cancer study, in which *PIK3CA* mutations were identified from plasma- and serum-derived cfDNA (61). Since then, several additional oncologic genotyping studies using CSF taken from neurosurgical resection or lumbar puncture (LP) have been reported (62–64).

In epilepsy genetics research, the analysis pipeline for cfDNA consists of CSF collection, with centrifugation to separate cells containing genomic DNA from the supernatant containing cfDNA (Figure 2c). Samples can be used directly or frozen. The cfDNA-enriched supernatant can then be used for NGS directly or subjected to targeted amplification via digital droplet PCR (ddPCR), with primers and probes designed to detect specific, preselected mutations. In 2021, the first use of cfDNA in epilepsy genetics was published by Ye and colleagues. cfDNA was extracted from 28 pediatric epilepsy patients and 28 adult controls via LP or during open resection, respectively. Epigenetic analysis confirmed that the cfDNA was indeed of neural origin. This group also reported that epileptic patients had approximately eight times more cfDNA than their matched normal controls, permitting the detection of pathogenic brain-limited somatic variants in *LIS1*, *TSC1*, and *BRAF* in three patients. Although it is theorized that epileptic patients may have higher cfDNA concentrations due to increased neural apoptosis, the higher concentrations seen among epilepsy patients in this study are likely confounded by baseline differences in age and method of CSF collection between groups (65).

That same year, Kim and colleagues studied 12 MCD patients with known mosaic pathogenic variants as identified from previous analysis of resected tissue. CSF was obtained during open resection, and extracted cfDNA was subjected to targeted preamplification before ddPCR. Previously known, tissue-derived somatic mutations in *PIK3CA*, *BRAF*, *and SLC25A2* were identified for only three of 12 patients, suggesting that the analysis pipeline still needed significant optimization, especially for detection of ultra-low VAFs (66). Most recently, in 2023, Chen and colleagues published a case report of a boy living with megalencephaly capillary malformation (MCAP) syndrome, a disease entity associated with epilepsy. However, because this patient did not suffer from epilepsy himself, he was not a candidate for surgical resection nor accompanying tissue-based diagnostics. NGS using cfDNA from CSF as well as DNA from both skin fibroblasts and blood detected a pathogenic, gain-of-function *PIK3CA* mutation with a range of differing VAFs, the highest being in fibroblasts at 37.3% and the lowest in blood at 2.0% (67).

Compared to sEEG-derived DNA, using CSF to identify brain-limited somatic mutations in epilepsy is significantly more nascent. Nevertheless, it has the potential to expand access to and reduce wait times for molecular diagnostics. While some studies use CSF collected during open resection, detection of variants at this stage has no comparative advantage to tissue-based diagnosis and may be too late to be of great clinical significance. Hence, future studies should focus on CSF obtained from LPs in order to obtain brain-derived genetic material long before a patient undergoes definitive surgery. Additionally, because an LP is a bedside rather than an operative procedure, it, much like sEEG, is associated with minimal morbidity and mortality (68). However, unlike sEEG, it does not require operating room time nor the expertise of a comprehensive epilepsy center. Lastly, recent advances in cancer research have demonstrated that tumor cfDNA can be used to track tumor growth and assess treatment effectiveness over time (69). Such temporal analysis using cfDNA would be a boon in epilepsy management, especially when tracking postoperative outcomes or responses to changes in medical regimens.

Despite these potential advantages, because CSF flows freely throughout the entire central nervous system, it cannot detect somatic gradients like sEEG-based methods. Instead, CSF analysis can simply identify a specific brain-limited mutation but cannot provide information about different VAFs across brain regions. Additionally, because ddPCR relies on probes and primers designed for a specific target sequence, ddPCR-dependent methods can only confirm suspected variants. Unlike sEEG-based techniques, CSF has minimal evidence for use in variant discovery. In fact, NGS methods using cfDNA have only been published in a single case report (67). Thus, CSF likely has more utility as a clinical screening tool for variants known to have particularly poor prognosis or for which targeted therapies have already been developed. All things considered, the most probable scenario is that these techniques are used in a complementary rather than mutually exclusive fashion. Since CSF often flows readily from the anchored bolts used to secure electrodes in the cranium during sEEG explantation, a paradigm in which electrodes and CSF are collected and analyzed simultaneously is certainly feasible.

## 4 Advantages and disadvantages of minimally invasive methods

### 4.1 Advantages of sEEG- and CSF-derived DNA

Beyond providing alternative substrates for genetic analysis, there are numerous specific advantages for sEEG- and CSF-derived genetic material (Table 1). As several studies have demonstrated, sEEG allows for more comprehensive sampling throughout the brain, especially of deep structures that are otherwise inaccessible surgically. It also provides for joint analysis of VAFs and electrophysiologic data to create maps of regional somatic mosaicism throughout the brain, which may help us understand how somatic mutations impact epilepsy pathogenesis (53, 56, 57). Furthermore, sEEG electrodes provide samples of normal brain tissue for each patient, allowing each to serve as their own intra-subject control. This is in comparison to resection-derived tissue, in which there is none or very little normal tissue available for comparative analysis. In the same vein, unlike with sEEG-based methods where spatial information for samples can be easily maintained, the anatomical orientation of bulk-brain samples is often lost in the process of extracting tissue in the operating room, sectioning portions to be sent for histopathological studies, and transferring remaining portions to the lab for analysis.

**Table 1:** The properties of each source of genetic material (resection-derived tissue vs. sEEG electrodes vs. CSF) in terms of invasiveness, accessibility, and potential applications.

Category	Source of Genetic Material		
	Resection-Derived Bulk-Brain Tissue	sEEG-Derived Microbulk Tissue	CSF-Derived cfDNA
Level of Invasiveness	Open	Minimal	Minimal or Open
Clinical Conditions for Collection	Open resection in which resected tissue is intentionally collected	Performance of sEEG for surgical evaluation and proper collection of explanted electrodes	Successful LP, craniotomy for resection, or collection from sEEG bolt on explantation
Analysis Time Frame	Days to weeks (depending on sequencing workflow)	Days to weeks (depending on sequencing workflow)	Hours to days if ddPCR, days to weeks if sequencing
Availability Outside of Specialized Epilepsy Centers	Varies	Varies	Yes
Performed in Children 0 to 3 Years	Yes	Varies by practice setting and surgeon preference	Yes
Ability to Detect Region-Specific Somatic Mutations	Yes	Yes	No
Genetic Material Extracted	Genomic DNA	Genomic DNA	Cell-free DNA
	RNA	RNA	
Need for Post-Extraction Amplification	No	Varies	Varies
Ability to Discover New or Unexpected Variants	Yes	Yes	Varies

cfDNA, cell-free DNA; CSF, cerebrospinal fluid; ddPCR, digital droplet polymerase chain reaction; LP, lumbar puncture; sEEG, stereo-electroencephalography (stereo-EEG).

Both sEEG electrodes and CSF have the potential to provide an earlier genetic diagnosis than that afforded by analysis of resected tissue, which is often only available after several failed medical treatments and an open procedure. The benefits of an earlier diagnosis are many, including earlier enrollment in appropriate clinical trials, adjustments to care coordination or medications, more specific prognoses, and better neuropsychological outcomes for patients later in life (19, 70, 71). Additionally, several states have no level IV comprehensive pediatric epilepsy centers, and, of those that do, centers tend to be clustered in metropolitan areas (72). In this regard, CSF-derived cfDNA is especially promising because it does not require the resources or expertise of a comprehensive epilepsy center. Many hospitals will be able to perform an LP for rapid initial diagnostic workup even if the patient's care must then be escalated to a more specialized center for CSF sample processing, sEEG implantation, or other clinical management.

### 4.2 Disadvantages of sEEG- and CSF-derived DNA

There are also several limitations of these minimally invasive methods that must be acknowledged. When considering CSF-derived cfDNA, its utilization does not allow for the variant gradient determinations possible with sEEG or even with surgical resection in some isolated cases, limiting our characterization and understanding of the hypothesized mosaic pattern of mutational burden that exists throughout the brain. cfDNA is also currently unsuited for variant discovery applications, limiting its current utility to that of a screening tool for particular variants of clinical interest. In sEEG, the field has yet to rigorously determine the exact composition and anatomical origin of sampled cells from each electrode. Electrode-derived cells can also become contaminated by local immune or blood cells, diluting the neural cells of interest. It is additionally unclear why some electrodes have better DNA yield than others, and although amplification strategies continue to improve, WGA has been shown to cause greater sequencing errors and possible false positive variant calls (73). Further, sEEG is not uniformly performed in all patients. Those with a clear surgical target from imaging and clinical evaluation often proceed directly to surgery, while sEEG in young children presents unique challenges that may dissuade some centers from performing the procedure in children under three years of age altogether (74–76). Lastly, both sEEG electrodes and CSF yield small amounts of DNA that require significant expertise and infrastructure for proper collection, storage, and processing. The current need for research laboratories to facilitate sample processing and sequencing increases the costs of these technologies and restricts their current use to comprehensive epilepsy centers or other research institutions.

## 5 Envisioning the future of genetics research in epilepsy

In all, the future of epilepsy diagnostics and research is promising. Both sEEG and CSF provide more opportunities to achieve a genetic diagnosis, which will not only help tailor clinical management of the individual patient, but will also advance research towards precision therapies and, hopefully, a cure. It is unlikely that sEEG electrodes and CSF will entirely replace resection-derived bulk tissue as a source of genetic material for molecular analysis. Rather, the development of these minimally invasive techniques will provide additional tools to further precision diagnostics and precision medicine. In the near future, we anticipate that patients and their epilepsy care teams will have several similarly effective options for diagnostic workup that can then be tailored to that patient's particular needs and clinical situation. CSF and sEEG thus provide greater flexibility in epilepsy management to diagnose more patients more quickly.

One of the many benefits of the strong foundation of research derived from the collection of bulk-brain tissue over the past three decades is that several research and care consortiums have already been established with the goal of combating the difficulties created by the scarcity of brain tissue for analysis. Thus, for centers without the physical or human capital to directly process and analyze CSF or sEEG electrodes, several well-worn paths exist to transfer these samples to centers capable of doing so. For example, our center has already developed several such collaborations, receiving samples from institutions across the country and beyond. We will analyze these samples and return any diagnostic results, in hopes of solving more genetic epilepsy cases and advancing pediatric epilepsy research. Though the main outputs of these research efforts to date have been improvements in the clinical management of epilepsy, we have hope that our combined and interdisciplinary efforts in this field will lead to surgical advancements as well. As we learn more about the role brain-limited somatic mutations play in driving epileptic networks, we anticipate a future in which neurosurgeons are able to deliver targeted gene therapies to brain regions with high mutational burden that are suspected to be driving epileptogenesis. Similarly, we envision a future in which somatic variant mapping informs surgical planning. Just as we aim for negative anatomical margins in tumor resection, we hope that research will aid in the establishment of genetic margins, thus developing a surgical approach that targets and removes regions with mutational VAFs above a critical threshold associated with epileptogenicity.

## 6 Conclusions

Molecular genetic analysis of resection-derived bulk-brain tissue has been critical to our current understanding of epilepsy genetics. However, CSF and sEEG electrodes have emerged as new means of expanding our access to brain-derived DNA, especially for patients who are not surgical candidates or who opt for minimally invasive surgical interventions. As these technologies mature, both methods can provide earlier and greater access to comprehensive genetic diagnostics for patients. In particular, sEEG allows for more comprehensive brain sampling as compared to bulk-brain tissue, permitting further investigation of somatic mosaicism's role in epileptogenesis. Although the wide use of both CSF- and sEEG-derived DNA remains constrained both by cost and the need for research laboratory resources, there is tremendous promise for these technologies in the near future to give patients more options for precision diagnosis and treatment. These minimally invasive techniques also give researchers more substrate for study and more opportunities to advance the field of epilepsy genetics.
